# Effect of four different forms of high intensity training on BDNF response to Wingate and Graded Exercise Test

**DOI:** 10.1038/s41598-021-88069-y

**Published:** 2021-04-21

**Authors:** Eugenia Murawska-Ciałowicz, Gilmara Gomes de Assis, Filipe Manuel Clemente, Yuri Feito, Petr Stastny, Jolanta Zuwała-Jagiełło, Bartosz Bibrowicz, Paweł Wolański

**Affiliations:** 1 Physiology and Biochemistry Department, University School of Physical Education, Wrocław, Poland; 2 Department of Molecular Biology, Gdansk University of Physical Education and Sport, Gdańsk, Poland; 3 Mossakowski Medical Research Centre, PAN, Warsaw, Poland; 4 Escola Superior Desporto E Lazer, Instituto Politécnico de Viana Do Castelo, Viana do Castelo, Portugal; 5 Instituto de Telecomunicações, Delegação da Covilhã, Covilhã, Portugal; 6 Department of Exercise Science and Sport Management, Kennesaw State University, Kennesaw, USA; 7 Faculty of Physical Education and Sport, Charles University, Prague, Czech Republic; 8 Department of Pharmaceutical Biochemistry, Wroclaw Medical University, Wrocław, Poland; 9 Polish Strength & Conditioning Association, Wrocław, Poland

**Keywords:** Biochemistry, Molecular biology, Neuroscience, Physiology

## Abstract

This study examined the effects of a nine-week intervention of four different high-intensity training modalities [high-intensity functional training (HIFT), high-intensity interval training (HIIT), high-intensity power training (HIPT), and high-intensity endurance training (HIET)] on the resting concentration of brain-derived neurotropic factor (BDNF). In addition, we evaluated the BDNF responses to Graded Exercise Test (GXT) and Wingate Anaerobic Test (WAnT) in men. Thirty-five healthy individuals with body mass index 25.55 ± 2.35 kg/m^2^ voluntarily participated in this study and were randomly assigned into four training groups. During nine-weeks they completed three exercise sessions per week for one-hour. BDNF was analyzed before and after a GXT and WAnT in two stages: (stage 0—before training and stage 9—after nine weeks of training). At stage 0, an increase in BDNF concentration was observed in HIFT (33%; p < 0.05), HIPT (36%; p < 0.05) and HIIT (38%; p < 0.05) after GXT. Even though HIET showed an increase in BDNF (10%) this was not statistically significant (p > 0.05). At stage 9, higher BDNF levels after GXT were seen only for the HIFT (30%; p < 0.05) and HIIT (18%; p < 0.05) groups. Reduction in BDNF levels were noted after the WAnT in stage 0 for HIFT (− 47%; p < 0.01), HIPT (− 49%; p < 0.001), HIET (− 18%; p < 0.05)], with no changes in the HIIT group (− 2%). At stage 9, BDNF was also reduced after WAnT, although these changes were lower compared to stage 0. The reduced level of BDNF was noted in the HIFT (− 28%; p < 0.05), and HIPT (− 19%;p < 0.05) groups. Additionally, all groups saw an improvement in VO_2max_ (8%; p < 0.001), while BDNF was also correlated with lactate and minute ventilation and selected WAnT parameters. Our research has shown that resting values of BDNF after nine weeks of different forms of high-intensity training (HIT) have not changed or were reduced. Resting BDNF measured at 3th (before GXT at stage 9) and 6th day after long lasting HITs (before WAnT at stage 9) did not differed (before GXT), but in comparison to the resting value before WAnT at the baseline state, was lower in three groups. It appears that BDNF levels after one bout of exercise is depended on duration time, intensity and type of test/exercise.

## Introduction

It is well established that exercise is beneficial to the health and functioning of the body^1^. Moreover, exercise results in beneficial adaptive changes (remodeling) observed at many levels, including molecular systems^2,3^. This ‘remodeling’ promotes the improvement of general physical fitness as well as the efficiency of energy processes, increases muscle mass, and improves neuromuscular coordination, which has a positive effect on brain function^4^. In addition, cognitive and memory-related functions are improved thanks to physical effort^5^.

Substances secreted by various cells are responsible for tissue remodeling. After entering the bloodstream, they participate in tissues/organs crosstalk. These substances are classified as growth factors, with multidirectional activity, affecting many essential biological processes^6^. These auto-, para- and hemocrine compounds are secreted by the skeletal muscles (myokines), adipose tissue (adipokines) or nervous system cells (neurokines)^7–10^. An example of such substances, which can communicate through different organs is brain derived neurotrophic factor (BDNF)^8^.

BDNF is a member of neurotrophic factors family and acts via the specific tyrosine kinase receptor B (TrkB)^11^. According to Rasmunsen et al.^12^ BDNF is widely expressed and produced in the brain. BDNF is also a contraction inducible protein^13,14^. Its expression is also observed in immune cells^15^ and endothelium cells^16^. BDNF crosses the blood-brain barrier in both directions and communicates with different brain structures, skeletal muscle, adipose tissue or immune system while taking part in homeostasis maintenance^17–19^. Although BDNF is mainly expressed and secreted in the brain, it circulates in platelets, which serve as the main storage of BDNF in the blood^20^. BDNF plays a significant role in neurogenesis by stimulating neuronal plasticity and facilitating neurons development, differentiation and survival. In addition, it takes part in synaptogenesis and dendritogenesis^20,21^. Moreover, BDNF participates in hippocampus cells differentiation, strengthening signal transmission inducing and maintaining long-term potentiation (LTP) of synaptic enhancement^22,23^, which are the molecular bases of cognition, emotional processes, spatial orientation or learning^5,20–27^.

Matthews et al.^13^ reported that the mRNA of BDNF and the protein expression were increased in skeletal muscles after exercise, yet BDNF was not secreted into the circulation. It is thought that BDNF produced in skeletal muscles is utilized locally during muscle’s fibers and nerve regeneration as a supporting factor of motor neurons’ survival, and promotor of growth of motor and sensory neurons^27^. It participates also in regulation of satellite cells differentiation and skeletal muscles regeneration^28,29^.

According to Pedersen et al.^30^ the main source of this myokine in skeletal muscles are probably neurons. BDNF and its receptor have a key role in central regulation of the energy balance and several reports suggest the possibility that the BDNF/TrkB axis in adipose tissue may have a role in the regulation of systemic metabolism^19,31–33^. BDNF via AMP- activated protein kinase (AMPK) is capable to enhance fatty oxidation^13,19,30^. In addition, it is reported that BDNF acts in an autocrine or paracrine fashion with strong influence on energy metabolism and plays important role in fat oxidation^33^. In addition, it is believed to also regulate weight loss, appetite suppression and modifiy the size of adipose tissue through a central mechanism^19,30^. According to Sornelli et al.^31,33^ BDNF is a new adipokine that is expressed in both white and brown adipose tissue in mice and rats during experimental stress and in type 1 diabetes.

Physical activity is known to provide many health benefits and is thought to be as a strong stimulus of brain health by increasing blood supply resulting in improvement of brain function, and prevention of nervous system diseases^4,8–10,30,34–37^. Physical exercise is a kind of activity that requires the brain to constantly monitor movement patterns, especially at the stage of learning of new motor activities^9,34,37^. BDNF is secreted, among others under the influence of physical exercise and is implicated in satellite cells stimulation that play an important role in muscle regeneration^28,29^. Different molecular mechanisms have been proposed to explain how aerobic exercise can impact BDNF synthesis in brain and peripheral tissue^34–39^.

High intensity functional training (HIFT) has gained a significant following over the last several years^40^. HIFT is multimodal type of training that includes multi-joint movements to create movement patterns that are consistent with everyday movements, and commonly known as “functional movements,” which engage the entire kinematic chain. These movements are performed as quickly as possible with the workload adjusted to the individual’s abilities, in a limited time period or with a limited number of repetitions, with the main goal to target different fitness domains – cardiorespiratory endurance, muscle strength, speed, coordination or anaerobic power, agility, flexibility^40–43^.

High-intensity interval training (HIIT) include short and intense elements of work alternated with low-intensity recovery periods. According to Buchheit and Laursen^44^, HIIT can include repeated short (<45 s) to long (2–4 min) bouts of high- but not maximal-intensity exercise, or short (≤10 s) repeated-sprint sequences [RSS] or long (20–30 s, sprint interval session [SIT]) all-out sprints, interspersed with recovery periods. These varying-length efforts combine to create training sessions that last a total of 5–40 min. Time of workouts is depends most of all on a participant's current fitness level as well as of the session intensity. The main goal of HIIT is to improve cardiorespiratory and metabolic function, as well as overall physical performance.

High intensity power training (HIPT) is a variation of the more popular HIIT, and incorporates high intensity resistance training by using varied multiple – joint movements and focused on high power output. In this type of training there is no defined recovery period and incorporates functional lifts such as the squat, deadlift, clean, snatch, and overhead press. The main goal of HIPT is to shape skeletal muscle mass, yet it can also be used as a stimulus for cardiorespiratory fitness and body composition^45,46^.

High intensity endurance training (HIET) is an endurance type of training with the intensity of 85–100% HR_max_. The time of this type of training is proportional for its intensity. Taking into account the age of athletes and physical fitness, HIET lasts from several minutes up to several hours^47–49^ and it is considered a continuous form of exercise. The best cardiorespiratory effects are observed when the intensity is about 85% VO_2max_ (~ 90% HR_max_). Continuous endurance training may be realized with long, slow distance (LSD) and long, high distance (LHD) or variable intensity. LSD training is characterized by constant pace of low to moderate intensity over an extended distance or duration. Heart rate varies in range 60 – 80% HR_max_ (~140 – 160 bpm). The goal of LSD method is energy expenditure maximizing and reduction of body mass by fat mass utilization. The intensity of LHD method is high and varies between 85 and 95% HR_max_. The volume of this type of training is low. The main goal of endurance training with high intensity is to shape the cardiorespiratory fitness by effective improving maximal oxygen uptake in individuals^49^.

Several studies have reported BDNF levels in blood increase after one bout of exercise^12,34–37,50^. Both acute and chronic exercise stimulate BDNF production and improve memory and mood^3,26,34,36,37,50–52^. Huang, et al.^53^ showed a correlation between VO_2max_ and the magnitude of BDNF changes. According to Schmidt-Kassow et al.^54^ and Rojas Vega et al.^50^, BDNF concentrations increases with the duration of exercise and return to baseline after few minutes of recovery. Saucedo Marquez et al.^55^ and Renteria et al.^56^ observed much higher levels of BDNF after a single session of HIT than in continuous exercise. Additionally, Yarrow et al.^57^ and Rojas Vega et al.^58^ reported an increase in BDNF after resistance training, while Goekint et al.^59^ did not observe BDNF changes either after an acute bout or after a 10-week strength training intervention. In another study, Correira et al.^60^ reported that acute strength exercise did not induce alterations in the BDNF level among healthy individuals.

Despite the number of studies demonstrating increases in BDNF secretion as a result of an exercise sessions and/or exercise training program, there are several studies opposing these reports. Rojas Vega et al.^50^ reported that BDNF during recovery was reduced in comparison to resting values. Figueiredo et al.^61^ also reported lower BDNF levels after training compared to pre-training values. As well as Nafuji et al.^62,63^ who reported lower levels of BDNF among trained athletes. Hebisz, et al.^64^ showed that BDNF did not change after six-months of SIT and decreased after intensive sprint interval exercise test (SIET). Murawska–Ciałowicz et al.^65^ did not report changes in resting BDNF after three-months of CrossFit training among men. As a result of these findings scientists believed that the type of exercise program may be a decisive factor in altering BDNF levels, as well as intensity of the exercise or the individual level of physical performance and physiological adaptation^62,63^.

Considering the contradictory accounts in the literature and taking into account the insufficient reports about the influence of different types of HIT on BDNF secretion, and the limited reports describing the BDNF response to standard aerobic and anaerobic performance tests, it would be of significant interest for practitioners to explain the dynamics of BDNF changes after several weeks of high-intensity training. To the best of our knowledge, no other study has compared changes of BDNF level after different types of HIT over nine-weeks among men of similar fitness levels.

Therefore, the aim of the study was to determine (1) if resting BDNF concentration change after nine-weeks of high intensity training of various types –HIFT, HIPT, HIIT and HIET; (2) examine BDNF changes after graded exercise test (GXT) and Wingate anaerobic test (WAnT); and (3) changes in performance and anthropometric parameters after nine-weeks of different types of HIT.

## Results

Prior to the experiment there were no differences in anthropometrical parameters noted among all measured groups (Table 1).

**Table 1:** Anthropometric characteristics of volunteers before the experiment.

Groups	Age (years)	Weight (kg)	Height (cm)	BMI (kg/m^2^)	FMI (kg/m^2^)	WHR
HIFT	26.9 ± 4.20	83.12 ± 7.30	1.78 ± 0.04	26.19 ± 2.48	4.58 ± 0.93	0.87 ± 0.04
HIPT	28.9 ± 3.70	79.93 ± 6.78	1.81 ± 0.07	24.48 ± 2.03	4.96 ± 1.46	0.88 ± 0.03
HIIT	26.5 ± 3.30	83.99 ± 6.50	1.81 ± 0.06	25.59 ± 4.38	3.81 ± 0.65	0.85 ± 0.06
HIET	28.9 ± 3.10	81.42 ± 6.50	1.77 ± 0.04	26.07 ± 2.04	4.31 ± 1.03	0.91 ± 0.02

Data are presented as mean ± SD.

*BMI* body mass index; *FMI* fat mass index; *WHR* waist to hip ratio.

After nine-weeks of training a significant reduction of fat mass (kg) and percentage of body mass were observed in all groups (Table 2). The greatest changes were noted in the HIIT group (18.5%) with the other groups changing as follows: 16.2% in HIFT group, 10% in HIPT and in endurance group in 10.8%. Changes in absolute LBM (kg) were not observed (Table 2), however, when expressed as a percentage of body mass the differences between the two stages were noted.

**Table 2:** Changes of FAT (kg and %) and LBM (kg and %) mass at stage 0 and 9 in all groups.

Groups	FAT (kg) Stage 0	FAT (kg) Stage 9	*p*	FAT (%) Stage 0	FAT (%) Stage 9	*p*	LBM (kg) Stage 0	LBM (kg) Stage 9	*p*	LBM (%) Stage 0	LBM (%) Stage 9	*p*
HIFT	14.48 ± 3.47	12.14 ± 3.56	0.02	17.27 ± 3.00	14.67 ± 3.09	0.001	68.65 ± 4.85	69.63 ± 5.03	0.102	82.73 ± 3.00	85.33 ± 3.09	0.001
HIPT	12.36 ± 2.25	11.12 ± 1.39	0.02	15.47 ± 2.55	14.02 ± 1.78	0.091	67.58 ± 6.2	68.44 ± 5.71	0.219	84.53 ± 2.55	85.98 ± 1.70	0.086
HIIT	16.39 ± 6.88	13.36 ± 5.31	0.02	18.64 ± 5.38	15.60 ± 4.13	0.001	67.60 ± 9.77	68.97 ± 9.51	0.176	81.36 ± 5.38	84.40 ± 4.13	0.001
HIET	13.48 ± 3.62	12.03 ± 2.34	0.04	16.45 ± 3.68	14.85 ± 2.47	0.087	67.89 ± 5.19	68.83 ± 5.24	0.198	83.47 ± 3.74	85.15 ± 2.47	0.088

Data are presented as mean ± SD.

**Figure 1:**
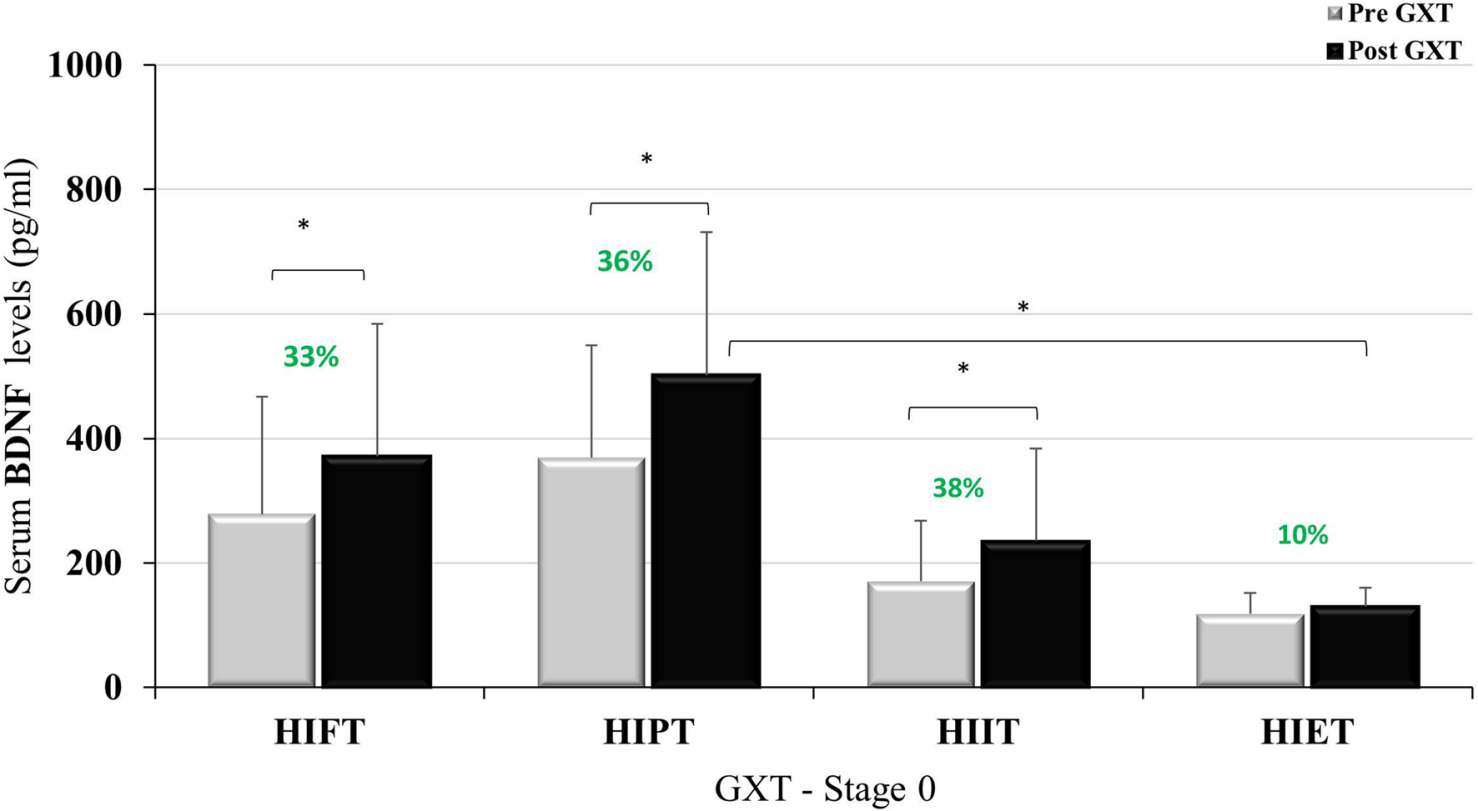
BDNF level at baseline, pre- and post GXT; *p < 0.05.

**Figure 2:**
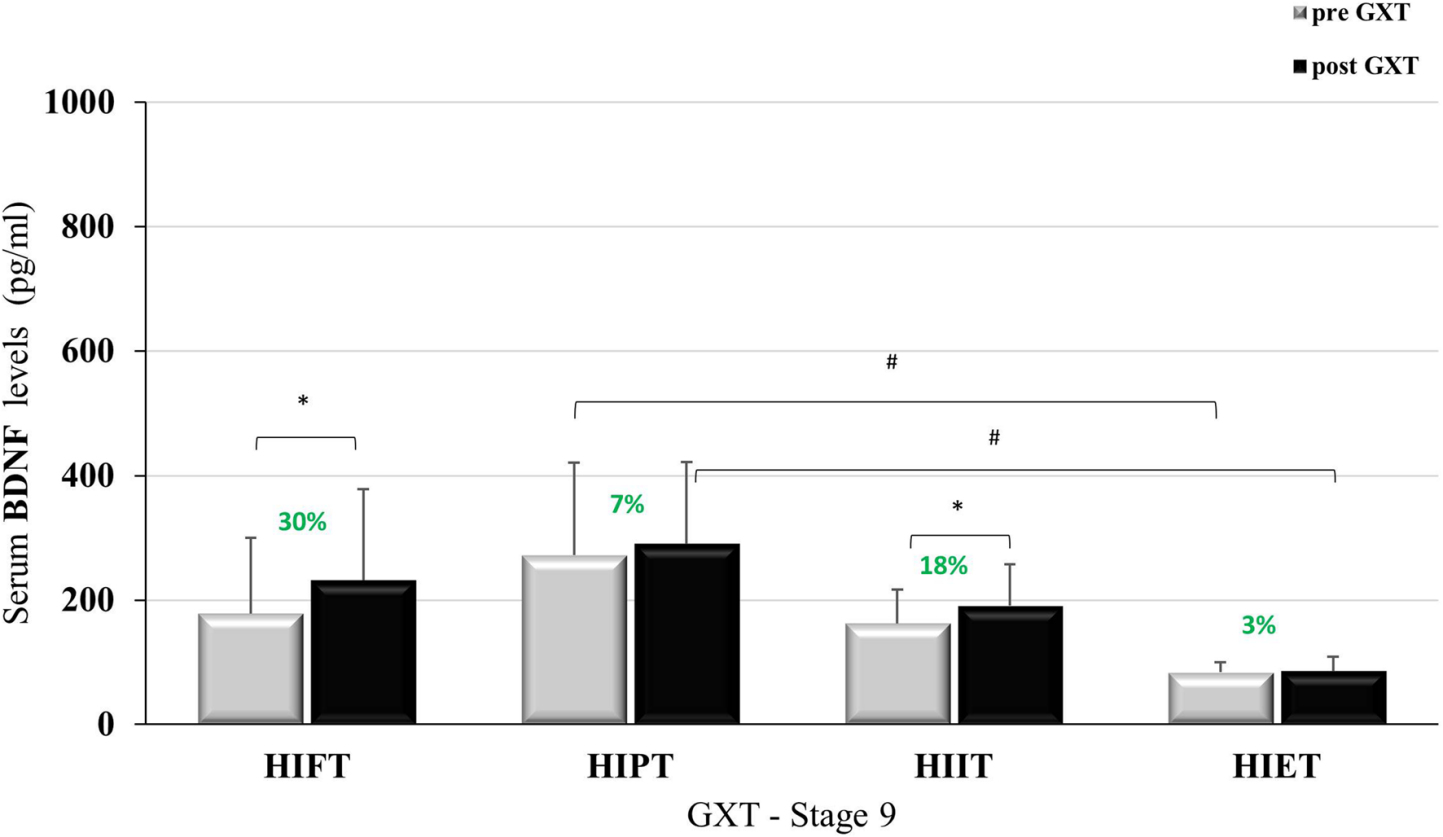
BDNF level after nine weeks training, pre- and post GXT; ^#^p < 0.01; *p < 0.05.

**Figure 3:**
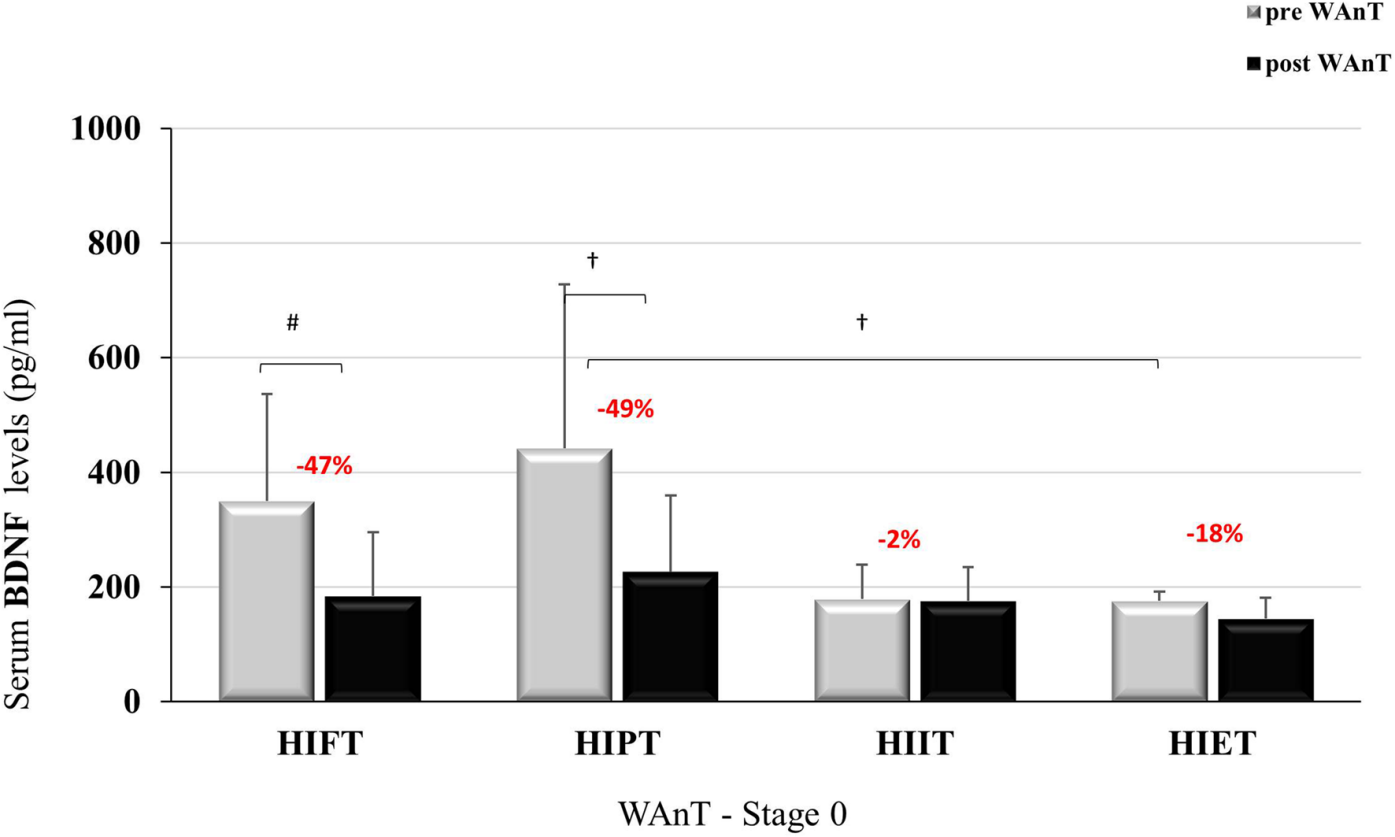
BDNF level at stage 0, -pre and post-WAnT; *p < 0.05; ^#^p < 0.01; ^†^p < 0.001.

**Figure 4:**
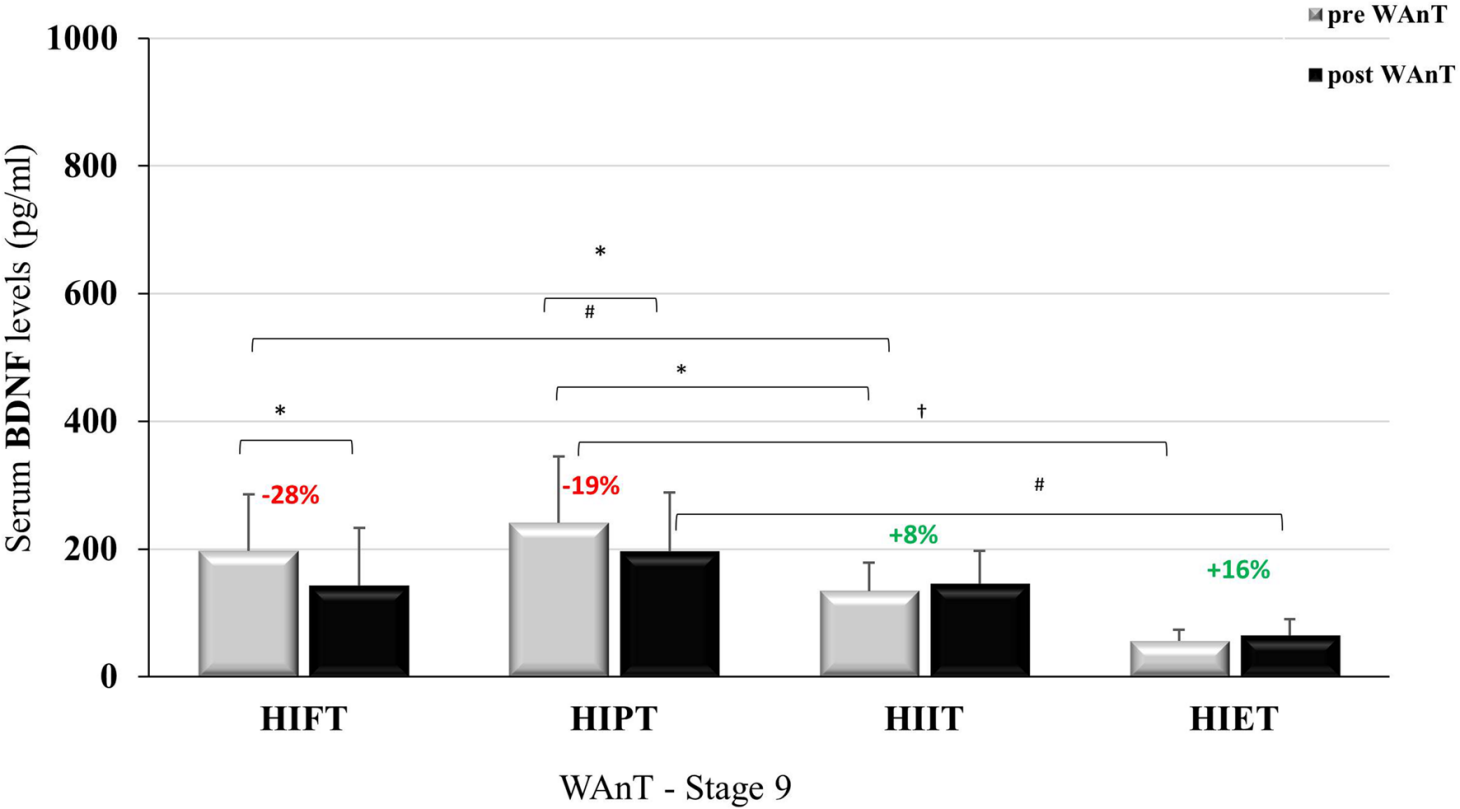
BDNF level in stage 9, -pre and post-WAnT; *p < 0,05; ^#^p < 0,01; ^†^p < 0,001.

After the nine-week intervention VO_2max_ improved in all groups except in the HIET group (Table 3). Additionally, the nine-week intervention had no impact on resting BDNF levels before the GXT. The levels of BDNF did not change after this time or was lower whencompared to the rest value before WAnT in the stage 0 (Table 3). The results of BDNF before and after the GXT and WAnT at baseline and after nine-weeks of training are presented in Figs. 1, 2, 3 and 4.

**Table 3:** VO_2_max and resting value of BDNF before GXT and WAnT at the baseline and after the 9 weeks.

	Resting BDNF					
	VO_2max_ (ml/kg/min)		BDNF (pg/ml) GXT		BDNF (pg/ml) WAnT	
	Baseline (Stage 0)	9 weeks (Stage 9)	Baseline (Stage 0)	9 weeks (Stage 9)	Baseline (Stage 0)	9 weeks (Stage 9)
HIFT	46.89 ± 5.79	50.97 ± 5.43**	278.99 ± 142.7	178.30 ± 121.7	349.96 ± 186.2	196.83 ± 89.38*^#^
HIPT	47.85 ± 3.73	50.77 ± 3.60*	369.26 ± 241.2	272.03 ± 148.9^#^	441.65 ± 285.4^†^	241.02 ± 104.6*^#†^
HIIT	44.81 ± 3.81	49.89 ± 5.62**	170.50 ± 97.46	162.23 ± 54.76	178.55 ± 60.41	134.19 ± 44.32
HIET	48.15 ± 5.99	50.81 ± 6.48	118.46 ± 33.92	83.80 ± 15.79	175.43 ± 76.18	55.74 ± 17.84*

Data are presented as mean ± SD.

*p < 0.05; **p < 0.01 in comparison to the baseline values.

^#^p < 0.05 in comparison to HIET.

^†^p < 0.05 in comparison to HIIT and HIET.

Figure 1 shows the BDNF concentrations at baseline, before and after GXT. No statistically significant differences were observed between the groups for resting BDNF concentration (F = 2.267; p = 0.101; η^2^ = 0.185) while after the GXT the differences in BDNF concentration between the groups were statistically significant (F = 3.389; p < 0.05; η^2^ = 0.253). In addition, statistically significant differences were observed within the groups before and after the GXT (F = 17.39; p < 0.001; η^2^ = 0.367), without noting significant differences between the groups when analyzing the effect size of the BDNF changes before and after the GXT (F = 1.964; p = 0.141; η^2^ = 0.164).

Changes of BDNF concentration before and after GXT at stage 9 are shown in Fig. 2. The resting values were statistically significantly different between the groups (F=4.978; p<0.01; η^2^=0.332), as were the BDNF values after the GXT (F=5.672; p<0.01; η^2^=0.362). Statistical analysis also showed a difference in the results before and after the GXT (F=4.370; p<0.05; η^2^=0.127). BDNF in the HIPT group were higher than in the HIET group (p<0.01).

Figure 3 shows BDNF concentrations at stage 0, before and after the WAnT. Statistically significant differences in BDNF concentration were observed between the groups at rest (F = 4.888; p < 0.01; η^2^ = 0.328). After the WAnT, differences between the groups disappeared (F = 1.102; p = 0.364; η^2^ = 0.099). Comparing differences within groups showed differences in BDNF concentration before and after the WAnT (F = 28.686; p < 0.001; η^2^ = 0.489). The magnitude of these changes was statistically significant (F = 7.270; p < 0.001; η^2^ = 0.421). The reduction of BDNF concentration after the WAnT in the HIFT group (↓ 47%) and in the HIPT group (↓ 49%) were significantly greater than in the HIIT group (↓ 2%) and HIET (↓ 18%).

Figure 4 presents BDNF concentration in stage 9 before and after WAnT. Statistically significant BDNF concentration differences were observed between the groups at rest (F = 10.087; p < 0.001; η^2^ = 0.50), as well as after WAnT (F = 4.849; p < 0.01; η^2^ = 0.327). Significant BDNF concentration differences were observed within the groups before and after WAnT, (F = 7,796; p < 0.01; η^2^ = 0.206). BDNF concentrations after WAnT in the HIFT group decreased (↓28%) and were statistically significantly higher than in the HIET group.

When analyzing the entire sample at the stage 0, a noted negative correlation between BDNF and FAT (%) was observed. A positive correlation was seen between BDNF with LBM (%) before and after the GXT and WAnT (Table 4). In addition, at stage 9 a positive correlations between BDNF level before and after GXT was observed, as well as with lactate before and after WAnT (Table 5).

**Table 4:** BDNF correlation with LA and anthropological parameters at stage 0 (Person’s coefficient).

Groups	LA After GXT	LA After WAnT	FAT (%)	LBM (%)	FAT (kg)	LBM (kg)
BDNF before GXT	0.1411	–	− 0.3035	0.3050	− 0.2353	0.0656
	p = 0.426	–	p = 0.081	p = 0.079	p = 0.180	p = 0.712
BDNF after GXT	0.2019	–	− 0.3876	0.3896	− 0.3105	0.0571
	p = 0.252	–	p = 0.024	p = 0.023	p = 0.074	p = 0.749
BDNF before WAnT	–	0.1321	− 0.3689	0.3709	− 0.2744	0.1906
	–	p = 0.457	p = 0.032	p = 0.031	p = 0.116	p = 0.280
BDNF after WAnT	–	0.1908	− 0.4146	0.4172	− 0.3145	0.0820
	–	p = 0.280	p = 0.015	p = 0.014	p = 0.070	p = 0.645

**Table 5:** BDNF correlation with LA and anthropological parameters at stage 9 (Person’s coefficient).

Groups	LA After GXT	LA After WAnT	FAT(%)	LBM (%)	FAT (kg)	LBM (kg)
BDNF before GXT	0.5398	–	− 0.3060	0.3060	− 0.2287	0.0333
	p = 0.001	–	p = 0.078	p = 0.078	p = 0.193	p = 0.852
BDNF after GXT	0.3618	–	− 0.1251	0.1251	− 0.0921	− 0.0325
	p = 0.035	–	p = 0.481	p = 0.481	p = 0.605	p = 0.855
BDNF before WAnT	–	0.3821	− 0.1473	0.1473	− 0.1001	0.0439
	–	p = 0.026	p = 0.406	p = 0.406	p = 0.573	p = 0.805
BDNF after WAnT	–	0.4438	− 0.2259	0.2259	− 0.1751	− 0.0232
	–	p = 0.009	p = 0.199	p = 0.199	p = 0.322	p = 0.896

We also noted a correlation of BDNF before GXT with VE_max_ after 9 weeks of training (Table 6). After the training it was noted that BDNF correlated with WAnT parameters. The positive correlation of BDNF with P_max_ and negative correlation with T2 time of P_max_ maintenance (Table 7) was noted.

**Table 6:** Correlation BDNF with VO_2max_ and VE_max_ measured in GXT parameters at stage 0 and stage 9.

Groups	VO_2max_ (ml/kg/min) Stage 0	VO_2max_ (ml/kg/min) Stage 9	VO_2max_ (ml/min) Stage 0	VO_2max_ (ml/min) Stage 9	VE_max_ (L/min) Stage 0	VE_max_ (L/min) Stage 9
BDNF before GXT	0.1859	0.2268	0.1037	0.1376	0.2021	0.4739
	p = 0.293	p = 0.197	p = 0.560	p = 0.438	p = 0.252	p = 0.005
BDNF after GXT	0.2333	0.1334	0.1027	0.0532	0.1515	0.3077
	p = 0.180	p = 0.452	p = 0.563	p = 0.765	p = 0.392	p = 0.077

**Table 7:** Correlation BDNF with WAnT parameters before and after 9 week training.

Groups	P_max_ (W) Stage 0	P_max_ (W) Stage 9	P_max_ (W/kg)Stage 0	P_max_ (W/kg) Stage 9	T1 (s) Sage 0	T2 (s) Stage 9
BDNF before WAnT	0.3119	0.3135	0.2887	0.4100	0.2926	− 0.3623
	p = 0.073	p = 0.071	p = 0.098	p = 0.016	p = 0.093	p = 0.035
BDNF after WAnT	0.1099	0.2134	0.2255	0.3769	0.2814	-0.3981
	p = 0.536	p = 0.226	p = 0.200	p = 0.028	p = 0.107	p = 0.020

The intensity of each training session was monitored by HR measurement, energy expenditure (EE) of the work during the main part and lactate level (LA) measured 10 min after the session completion. The average values of these parameters for all sessions are presented in Table 8. Moreover, in this table the average internal load for all sessions evaluated based on the volunteers RPE is presented.

**Table 8:** Markers of training intensity and internal load.

	Lactate (mmol/L)	EE (kcal/min)	HRmax (b/min)	%HRmax (%)	RPE (AU)
HIFT	9.94 ± 2.42^†^	12.52 + 1.82^†^	179.67 ± 11.75	92.76 ± 5.46	958.00 ± 87.53^#^
HIPT	4.07 ± 2.02*	9.57 ± 1.97*	166.06 ± 13.54*	88.31 ± 5.55*	1013.20 ± 66.53
HIIT	12.79 ± 3.42	10.27 ± 1.60	184.55 ± 8.12	95.30 ± 52.64	1001.85 ± 51.69
HIET	11.21 ± 2.63	11.35 ± 1.09	180.19 ± 7.59	94.81 ± 2.43	1126.69 ± 58.31

Data are presented as mean ± SD of all sixteen sessions.

*p ≤ 0.01 in comparison to all groups.

^†^p ≤ 0.05 in comparison to all groups.

^#^ p ≤ 0.05 in comparison to HIET.

## Discussion

In this study we investigated the effectiveness of nine-weeks of several high-intensity training protocols on resting BDNF level. Moreover, we wanted to evaluate BDNF response to GXT and WAnT pre and post training intervention. Three variant directions of changes in BDNF serum concentration were observed.

Resting BDNF post training before GXT did not change in any of the groups. Before WAnT resting values were significantly lower in HIFT, HIPT and HIET in comparison to pre training values. Between GXT and WAnT two days of recovery were applied in both stages. The high fluctuation of BDNF levels has been observed in BDNF resting values before GXT and WAnT in stage 0; however, no significant differences were noted between both tests-days. In stage 9, differences in BDNF were statistically significant between groups before GXT and WAnT but without changes between both tests.

We speculated that it is an effect of individual variability^66^, or daily variation in BDNF secretion^67,68^. Several studies have already demonstrated that BDNF is implicated in the regulation of circadian pacemaker function in the central nervous system. The highest level of BDNF is noted in the morning (at 08:00 h) and decreasing throughout the day. Plasma BDNF levels is significantly lower at 12:00 h in comparison to BDNF levels in blood at 08:00 h^68^. perhaps the fluctuations in our BDNF values is due to diurnal variations as blood sample were collected between 08:00 h and 13:00 h.

In our study the BDNF response to GXT was not the same as it was noted for BDNF response to WAnT. The BDNF was increased after the GXT pre and post training intervention, although the magnitude of the change was lower post training in comparison to pre values. Before training, an increase of over 30% was recorded. At stage 0 the greater variation of BDNF between groups was also observed after GXT than in post training. In contrast to the result observed after GXT were results of BDNF after WAnT pre and post training intervention. In both stages BDNF decreased or did not change in comparison to the resting values. With this test, more intense changes were also found at stage 0 than in stage 9.

The improvement of VO_2max_ in all groups except the HIET we also observed. According to many studies very high-intensity workouts, as HIFT, HIIT^40,44,69–72^, provoke anaerobic metabolism, high lactate concentration and oxygen deficit. Such conditions can stimulate BDNF secretion which can be mediated by PGC-1α (peroxisome proliferator-activated receptor-gamma coactivator-1α) as well as by lactate. PGC-1α is a key regulator of BDNF secretion and lactate metabolism. Between PGC-1α and BDNF the positive loop exists^35,38,73^. Moreover lactate, is an energy fuel for brain^74^ as well as a key mediator of neuroplasticity and BDNF regulation^38,39,73^. It can induce PGC1α/FNDC5/BDNF pathway through the silent information regulator 1 (SIRT1) activation^38,75–77^. It is postulated that PGC-1α is also the main factor influencing the biogenesis of mitochondria and is highly expressed in tissues rich in mitochondria and active oxygen metabolism such as brain, brown adipose tissue or skeletal muscles^69,70^. Oxygen deficiency during exercise and increases in LA concentration promote mitochondrial biogenesis^70–73^. Unfortunately, despite maximal oxygen uptake improvement reported in our study we have not observed correlation between BDNF and VO_2max._ It is also interesting that in the HIET group the average of LA level after all sessions was the highest among training groups; however, no improvement in VO_2max_ was noted after 9 weeks. This evidence suggests that not only training length, intensity, but also interrupted type of modalities is important in maximal oxygen uptake modification.

Reduction or no BDNF level changes observed after WAnT or high intensity training were noted by several authors^62–64,78–80^. Hebisz et al.^64^ found no changes in BDNF at baseline, as well as after two and six months’ of SIT training. Figueiredo et al.^61^ also reported lower BDNF values after eight-weeks of HIET training combined with strength training. In our study, the HIET group had reduced resting BDNF levels after nine-weeks of training in comparison to baseline values. In another study it was observed that BDNF returned to baseline during recovery in a sedentary group while in the trained group were reduced below baseline^62^. Moreover, after a maximal test BDNF increase much more in sedentary group (30%) than in trained athletes (only 11%). This observation suggests that greater dynamics of changes of BDNF levels can be observed in sedentary participants in comparison to the physically active individuals^63^.

We can speculate several possible mechanisms in this BDNF reduction. One of them could be a body mass and fat mass. Glud et al.^81^ measured BDNF in obese or overweight men and women after physical training and recorded a reduction of BDNF in men and women. The significant reduction of body mass with subsequent decrease of BDNF level was also reported by Lee et al.^82^ after a 12-weeks program for weight reduction in overweight men. Taking into account our study included normal weight men, and we have observed a negative correlation between BDNF level and percentage of body fat, as well as the significant reduction in the body mass, it is quite possible that body fat mass may explain the lack of BDNF changes or reduction observed in our study. Lommatzsch et al.^83^ also reported that body weigh negatively during recovery BDNF levels. It could be explained by the physiological/metabolic stress during long lasting high intensity trainings. According to Sornelli et al^31,33^ BDNF is present in adipose tissue and has potential anorexigenic effects. Moreover, BDNF can act via PGC1α/FNDC5/BDNF pathway and stimulates irisin secretion during skeletal muscle contraction. Irisin is produced by fibronectin type III domain containing 5 (FNDC5) cleavage^75^ and is an exercise hormone capable of increasing energy expenditure by fat oxidation, browning of white fat tissue and promoting weight loss^35,38,84,85^.

Another possible explanation could be seasonal variations observed in BDNF secretion. Bus et al.^86^ and Molendijk^87^ observed a high correlation of BDNF with amount of sunlight. The nadir values of BDNF were noted in the early spring and the peak serum levels were observed in the early autumn. In our study, the first round of measurements (stage 0) were performed in the autumn (October/November) and the second round of measurement in early spring (February/March). In line with this seasonal variation in BDNF we can suppose that the weaker respond of BDNF after both tests post-training may be explained by this mechanism.

It is also postulated that cAMP-response element binding protein (CREB) is an important regulator of BDNF secretion^88,89^. However, CREB activity is under influence of serotonin, which is dependent on amount of light^90^, and is higher during long days. Thus, serotonin is the most essential players involved in BDNF signaling. According to Martinovich^91^ and Jin^92^ a specific synergy between BDNF and serotonin signaling systems exists where a feedback loop between the two molecules exist. As it was previously mentioned BDNF can cross blood-brain-barrier^12,19,21,91^. In line with this it could be supposed that the peripheral level of BDNF can reflect the BDNF level in the brain and any seasonal and circadian BDNF changes in brain can reflect the BDNF in periphery.

The other possible biological mechanisms related to the reduction of BDNF could be explained by usage and consumption of BDNF in the regeneration of nerve fibers and miofibers and the inflammatory process that could occur in damaged tissues. Skeletal muscle damage is often observed during strength training. Resistance exercises performed with high intensity as a part of HIFT, HIIT or HIPT training could provoke mechanical muscle damage especially in the eccentric phase of movement during weightlifting^93^. The training programs included in our study are considered very intense. The average intensity exceeded 90% HR_max_. It may be hypothesized that BDNF circulating in the blood was consumed by muscles in the process of repairing damaged structures and more intense neuroprotection.

According to several studies^94,95^ in several conditions associated with adrenergic stimulation, an increase in peripheral count of larger platelets has been observed. Such conditions are always present during exercise^95,96^. Exercise stimulates thrombocytosis and megakaryocytes release from the liver and spleen^95–99^. Platelets’ α-granules are the main BDNF storage site in the blood (~99%). Only a small amount of free BDNF circulates in the plasma^99,100^. Platelet’s α-granules contain many different growth factors. When an injury occurs, growth factors secretion by platelets and macrophages is induced and the inflammation-healing process is initiated^99,100^. According to Nofuji, et al.^63^ regular exercise facilitates the utilization of circulating BDNF after acute exercise with maximal intensity.

Metabolic/physiological stress can also decrease BDNF secretion. It has been reported that cytokines and chemokines are secreted in both central and peripheral nervous systems during psychological stress and that BDNF is thought to be involved in the neuroimmune axis regulation^101,102^. In addition, Jin et al.^90^ report that expression of BDNF is strongly affected by immune cells and the immune factors they secrete. It is well known that very intensive physical effort is a great stress for the body^103,104^. Increased stress levels affect BDNF mRNA and significantly reduce BDNF expression^69^. According to Rasmussen et al.^12^, 70–80% of BDNF circulating in the blood is produced in the brain both in restitution and after exercise. According to de Assis and Gasanov^105^ BDNF negatively correlates with the level of cortisol a catabolic hormone. Garcia- Suarez et al.^106^ also observed no changes of BDNF which was accompanied by higher level of cortisol and cortisol/BDNF ratio. Intensive training and the accompanying strong physiological stress reduce the concentration of testosterone with the associated increase in cortisol. It was also shown that testosterone administration increases BDNF protein levels in castrated male rats^107^. Unfortunately, we did not determine the concentration of hormones and cytokines, however, the level of lactate, energy expenditure, RPE and HRmax noted after each sessions always were high and may be evidence of high metabolic load/stress.

The genetic bases of our finding should also be considered. Human BDNF genes has been identified as a single nucleotide polymorphism (SNP). It results in valine (Val) for methionine (Met) substitution in position 66 in pre-pro-BDNF which is a precursor protein of BDNF^108^. It is quite possible that this mutation has important influence on BDNF probably by impairment of the secretion and function of this protein. According to Leraci et al.^109^ the BDNF Val66Met polymorphism impairs the beneficial behavioral and neuroplasticity effects induced by physical exercise and moderate the exercise response.

Even though several studies exist evaluating the influence of exercise on BDNF, to the best of our knowledge, this study is the first to compare BDNF changes as a result of four different high-intensity exercise programs. Even though no study is without limitations, we have attempted to reduce these by enlisting a homogenous participant pool, who exercised at the same facility, using the same equipment during testing and training sessions.

## Conclusions

Our research shows that very intensive forms of training with anaerobic metabolism lasting nine-weeks does not affect BDNF levels at rest and 10 min after WAnT. Exercise performed in anaerobic conditions reduces BDNF levels. Even though the mechanisms that explain these changes are not easily understood, we speculate that it can be connected with diurnal variation or seasonal changes negatively affected by secretion of cortisol and by anabolic/catabolic hormones disruption, which in turn leads to a significant decrease in BDNF concentration, which were not measured in this study. Therefore, future research should expand our findings to include hormonal and immunological aspects. As a results, our findings and their implications should be discussed in the broadest context possible.

## Materials and methods

### Study design

**Figure 5:**
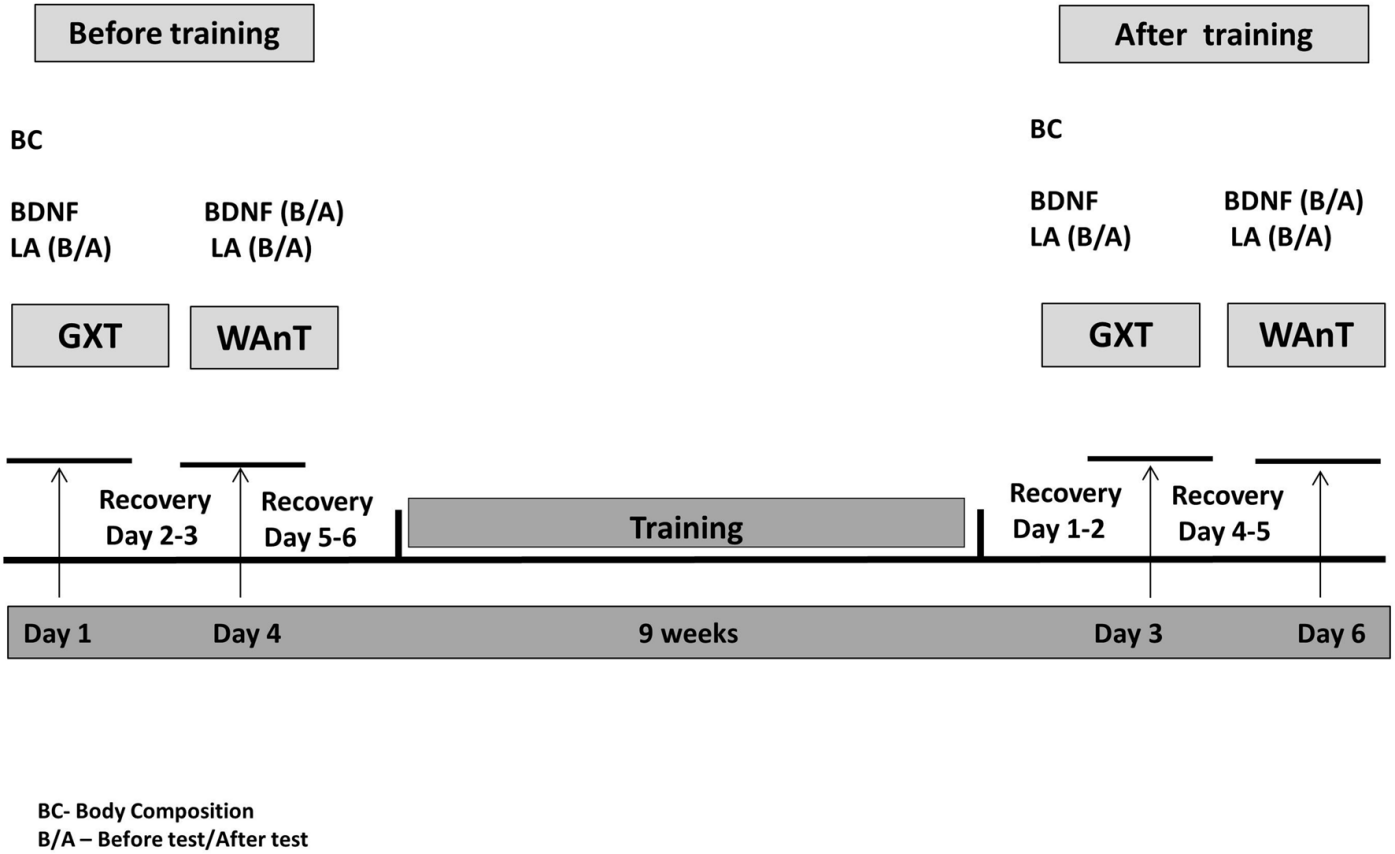
Scheme of GXT and WAnT tests before and after 9-week training.

A pre-post study design comparing the effects of four high-intensive interventions on the BDNF concentration, maximal oxygen uptake (VO_2max_) and anaerobic power was conducted. The interventions lasted nine-weeks with three sessions per week. Participants were randomly assigned to four groups. Before and after the intervention, participants were tested using a graded exercise test (GXT) with progressive intensity performed on a treadmill. The Wingate anaerobic test (WAnT) was also completed and performed on a cyclo-ergometer. BDNF concentration was assessed before and 10 min after the GXT and WAnT. Both measures were completed at baseline and after nine-weeks of trainings. The study design is presented in Fig. 5.

### Participants characteristic

The study was initiated with sixty men randomly classified into four groups (15 participants per group). However, during the course of the nine-weeks several participants withdrew as a result of injury (n = 8), and others as a result of the intensity of the program (n = 5). The study was designed to maintain the highest level of rigor and required participants to participate in the greatest number of sessions (no more than 10% absence was accepted). Seven individuals were excluded as a result of missing the baseline testing session. The final sample consisted of thirty-five men (27.80 ± 3.59 years; 25.55 ± 2.88 kg/m^2^ body mass index (BMI); 4.41 ± 1.14 kg/m^2^ fat mass index (FMI)], with participants divided into four training groups: HIFT (n = 8), HIPT (n = 9), HIIT (n = 9), HIET (n = 8). The inclusion criteria for the study included healthy man, between 20–35 years, and recreationally active.

Physical activity level was established based on the interview with the research team and on frequency of declared participation in the voluntary exercise per week (1–3/week about 1 h with low to moderate intensity (e.g., running, cycling, fitness gym, swimming, climbing). All participants engaged in physical only for fun and their own satisfaction, and were not considered athletes. All participants were in good health, and approved by the research team based on medical examinations. Study participants were approved to engage in HIT exercise after written agreement with a physician. Conversely, the exclusion criteria included: circulatory and respiratory system disorders, cigarette smoking, diabetes mellitus, thyroid diseases, hypertension, joint pain, and musculoskeletal injuries. Individuals who completed the study declared they only participated in the training classes provided by the research team and adhered to the training cycles. All participants were asked, and declared not to use the supplements, ergogenic aids and specific diets. They individually (for the personal use only) controlled their diet for energy and basic energetic elements.

Before testing and training, all participants were informed about the purpose of the tests, the procedures for performing biochemical and performance tests, and the possible effects during post—performance discomfort. A research protocol was presented to them and safety principles were discussed. Each participant provided informed written consent to participate in the study. Moreover, they also were informed that they could resign from the study at any stage without reason given. The research was conducted in accordance with the Declaration of Helsinki. The study was approved by and performed in accordance with the recommendation of the Bioethics Committee of Scientific Research at University School of Physical Education in Wrocław, Poland (resolution of 13/03/2017, No. 4/2017).

### Anthropometrics and body composition

Body composition and body mass were measured at baseline (stage 0) and after 9-weeks of training (stage 9) just before the GXT test. Body composition analysis was performed by use of a BodyMetrix BX 2000 device (*Intel Matrix, USA*). All procedures of body composition analysis were made in accordance with previously stablished protocols^110^. We measured fat mass (FAT) and percentage of body fat (%FAT) as well as lean body mass (LBM). Moreover, the BMI was calculated based on body mass (kg) and height (m) of volunteers. FMI was calculated based on the body fat mass (kg) and body height, and the waist-to-hip ratio (WHR) on the basis of circumferences of waist and hip (cm).

### Biochemical analysis

Participants were given a meal 2 h before GXT and WAnT. The blood samples for biochemical measurements were collected between 08:00 h and 13:00 h. Before each test and 10 min after the blood was collected from the basilic vein to determine BDNF concentration. The blood was than centrifuged and the serum was frozen at − 85 °C. When the serum from all volunteers had been collected all samples were thawed and BDNF was measured. Lactate level (LA) was collected from the fingertip and was also measured before and 10 min after both tests completion.

The Nori Human BDNF ELISA Kit (*Genorise, USA*) was used for the determination of BDNF concentration in the serum. Detection range of this method was 15–1000 pg/ml, sensitivity of this methods was 3 pg/ml, intra-assay coefficient was 5% and inter-assay coefficient was 9%.

The colorimetrical method was used for lactate concentration in the capillary blood. The Lactate Cuvette Test kit (*Dr Lange, Germany*) was used for this purpose and the Mini Photometer Plus LP20 (*Dr Lange, Germany*). The normal range of this parameter was established on 0.6–0.9 mmol/l.

The enzymatic amperometric methods with chip-sensor technology was used to evaluate the lactate level after each session completed in the fitness gym. The Lactate Scout 4 (*EFK Diagnostics for Life, UK)* was used for this purpose. Measuring range of this method is 0.5–40 mmol/L with inter assay coefficient ≤ 1.5%.

### Tests protocols

The Graded Exercise Test (GXT) and Wingate Anaerobic Test (WAnT) are very popular tests and very often used to physical performance evaluation. The GXT is used for maximal oxygen uptake (VO_2max_) measurement and the WAnT for maximal anaerobic power and capacity. The detailed procedures of GXT and WAnT tests were described previously^108^.

Briefly, the GXT was performed on a treadmill (*SEG-TA7720 treadmill InSportLine, Czech Republic*), with the initial belt speed set at 6 km/h, with 2 km/h increments every 3 min until exhaustion. All participants used a face mask using a one-way mask where the exhalated air was directed to a spirometer (Quark b^2^; *Cosmed, Italy*). Ventilatory parameters were recorded ‘breath by breath’ and then averaged every 30 s. VO_2max_ (ml/min/kg), respiratory exchange ratio (RER), maximal pulmonary ventilation (VE_max_) and lactate were also analyzed. The maximal oxygen uptake reaching in the GXT test ought to be established by the plateau in oxygen uptake. Because none of the volunteers met plateau criterion, the VO_2_max was confirmed by at list two end criteria: heart rate (HR) ≤ 10 b/min or ≤ 5% of age-predicted maximum (220-age); RER >1.00–1.15; blood lactate concentration after test ≥ 8 mmol/L^111,112^. During the entire test the HR was recorded using POLAR m400 sportester (*Kempele, Finland*). 10 min after the test completion the LA concentration was measured.

The WAnT was conducted using a Monark 828E cycloergometer (*Monark Lidingo, Sweden*). The procedure required participants to pedal as fast as possible for 30 s. The goal of the test was to generate the highest velocity possible and maintain it for the duration of the session (30 s). The external load was set at 7.5% of the individuals body mass. The warm-up before the test consisted of pedaling for 5 min with a 50W load. During the warm-up the heart rate should also correspond to 150 beats/min. During the test there are recorded such parameters as: maximum power (P_max –_ W; W/kg*),* time to reach maximal power (T1), time of maximal power maintenance (T2), minimal power (P_min_), index of fatigue (IF), total work (TW*—*kJ; J/kg*).* 10 min after the WAnT completion LA concentration was measured. Between GXT and WAnT two days of recovery was used in stage 0 and stage 9.

### Training protocols

The training protocols differed in the amount of intensity used and always included a general warm-up and a cooldown (Table 9). Each training session was monitored by HR using the Polar 400 data and energy expenditure and lactate measurements were evaluated 10-min after the session was completed. Participants evaluated their effort and were asked their exertion 30-min after the session using the ten points Borg Rating of Perceived Exertion (RPE) scale^113^. The score provided was then multiplied by the time of the session in minutes to determine the internal load of the session^114^. The answers were provided individually, and the participants were previously familiarized with the scale.

**Table 9:** General overview of training regimen.

Summary of TRAINING SESSION								
Groups	Warm-up	Work	Total work	Rest	Total rest	TC /EL	Cool-down	Σ time/session
HIPT	10 min	8 rep × 30 s	4 min	8 rep × 150 s	20 min	20 min	5 min	59 min
HIIT	10 min	6 rep × 90 s	9 min	6 rep × 90 s	9 min	20 min	5 min	53 min
HIET	10 min	12 min	12 min	–	–	20 min	5 min	47 min
HIFT	10 min	8 rep × 30 s	4 min	8 rep × 150 s	20 min	20 min	5 min	59 min

Summary of MICROCYCLE								
Groups	Warm-up (Σ)	Work	Total work	Rest	Total rest	TC /EL	Cool-down (Σ)	Σ time /week
HIPT	3 × 10 min (30 min)	3 × 4 min	12 min	3 × 20 min	60 min	3 × 20 min (60 min)	3 × 5 min (15 min)	177 min
HIIT	3 × 10 min (30 min)	3 × 9 min	27 min	3 × 9 min	27 min	3 × 20 min (60 min)	3 × 5 min (15 min)	159 min
HIET	3 × 10 min (30 min)	3 × 12 min	36 min	–	–	3 × 20 min (60 min)	3 × 5 min (15 min)	141 min
HIFT	3 × 10 min (30 min)	4 + 9 + 12 min	25 min	20 + 9 min	29 min	3 × 20 min (60 min)	3 × 5 min (15 min)	159 min

Summary of the 9 week MESOCYCLE								
Groups	Warm-up (Σ)	Work	Total work	Rest	Total rest	TC /EL	Cool-down (Σ)	Σ time/9 weeks
HIPT	9 × 30 min (270 min)	9 × 12 min	108 min	9 × 60 min	540 min	9 × 60 min (540 min)	9 × 15 min (135 min)	1593 min (26 h 33 min)
HIIT	9 × 30 min (270 min)	9 × 27 min	243 min	9 × 27 min	243 min	9 × 60 min (540 min)	9 × 15 min (135 min)	1431 min (23 h 51 min)
HIET	9 × 30 min (270 min)	9 × 36 min	324 min	–	–	9 × 60 min (540 min)	9 × 15 min (135 min)	1269 min (21 h 09 min)
HIFT	9 × 30 min (270 min)	9 × 25 min	225 min	9 × 29 min	261 min	9 s × 60 min (540 min)	9 × 15 min (135 min)	1431 min (23 h 51 min)

TC/LC—technique correction and learning of the exercises performed.

#### Protocol of HIPT training

In the HIPT group, all training sessions lasted 59 min, with the conditioning phase lasting 24 min (Table 9). For this training, all participant completed eight sets, each lasting 30 s, of strength exercises (Table 10). The load in each exercise was 75–95% 1RM (established as: weight/[(1.0278 − (.0278*reps)], the number of repetitions were from 3 to 12, and the intensity of the exercises performed was to be greater than 85% HR_max_. HR during training was measured by POLAR m400 sport tester. Each time after work, there was a 150-s break. Training units were completed as many rounds as possible (AMRAP). The main assumption of the training was to perform each repetition with maximum intensity. The total weekly microcyle time in the HIPT group was 177 min, and the 9-week mesocycle 26 h and 33 min. The training unit scheme corresponded to day 1 for the HIFT group.

**Table 10:** Type of exercises used during the main part of the training sessions.

**Exercise during training session of HIPT/CrossFit**			
Bench press	Box jumps with a load	Clean	CLEAN AND JERK
Deadlift	Front suat	Kettlebell clean	Kettlebell swings
Lunges with barbells	Overhead squat	Snatch	
**Exercise during training session of HIIT/ CrossFit**			
Box Jumps	Box jumps with a load	Burpees	Clean
Clean and jerk	Deadlift	Dips	double unders
Front squat	Jump rope	Kettlebell swings	Knees to elbows
Lunges	Lunges with barbells	Overhead squat	Push-ups
Rope climb	Row	Sit-ups	Toes to Bar
**Exercise during training session of HIET/CrossFit**			
Air squat	Box jumps	Box jumps with a load	Burpees
Clean	Clean and jerk	Dips	Double unders
Front squat	Hand stand push-ups	Jump rope	Kettlebell swings
Lunges	Lunges with barbells	Overhead squat	Push-ups
Rope climb	Row	Sit-ups	Toes to bar

#### Protocol of HIIT training

In the HIIT group, the training session lasted 53 min (Table 9), including the main part of the training of 18 min. It consisted of 6 sets each lasting 90 s, of exercise separated by a 90 s break. Endurance and strength exercises for the HIIT type were used here (Table 10). The load in individual exercises could not exceed 60% 1RM (established as: weight/[(1.0278 − (.0278*reps)], the number of repetitions was not determined, and the task of the person performing the exercise was to perform as many repetitions of the given exercise as possible during 90 s, or two exercises in a closed cycle with the number of repetitions given. The intensity of the performed exercises was to be greater than 85% HR_max_. Each time after work, there was a 90-s break. Training units were conducted, among others in the form of AMRAP and “You Go I Go”. The main assumption of the training was to perform each repetition with maximum intensity. The total weekly microcyle time in the HIIT group was 159 min, and the 9-week mesocycle 23 h and 51 min. The training unit scheme corresponded to the training environment from the HIFT group.

#### Protocol of HIET training

Training session in the form of HIET lasted 47 min and was the shortest among all the session in the project (Table 9). The main part of the unit was carried out in a continuous form for 12 min using endurance type of exercises (Table 10). The load in individual exercises could not exceed 40% 1RM, the number of exercises from 4 to 8, the number of repetitions from 10 to 20, and the intensity of performed exercises were to be greater than 85% HR_max_. Training units were conducted in the form of AMRAP. The main assumption of the training was to perform each repetition with maximum intensity. The total weekly microcyle time in the HIPT group was 141 min, and the 9-week mesocycle 21 h and 9 min. The training unit scheme corresponded to the Friday training from the HIFT group.

#### Protocol of HIFT training

**Figure 6:**
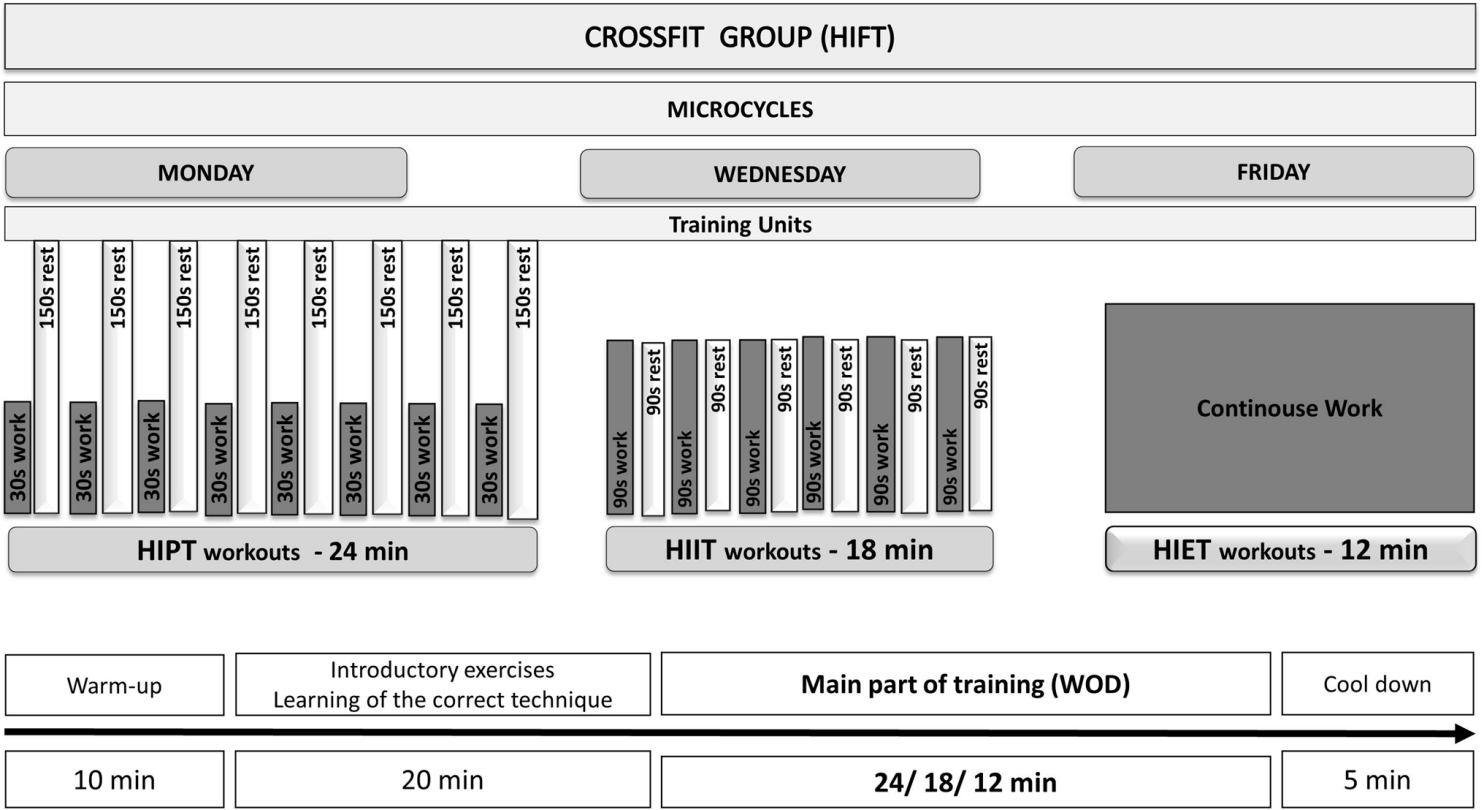
Scheme of HIFT microcyles.

In the HIFT group, the training units had a different duration depending on each training day of the training microcyle. On day 1 (Monday), classes were conducted in the form of HIPT training and lasted 59 min. The second classes in the training microcyle took place on Wednesday, were conducted in the form of HIIT training and lasted 53 min (Table 9). Training in the form of HIET lasted 47 min and was carried out as the last in a weekly microcyle (on Friday). The total weekly microcyle time in the HIFT group was 159 min, and the 9-week mesocycle 26 h and 33 min The scheme of workouts used for the HIFT group is presented on Fig. 6.

During the training, the participants of the HIPT group did the same exercises three times a week as the HIFT group during classes on Monday (Table 9). The HIIT group did the same exercises during each workout as the HIFT group did on Wednesdays, while the HIET group did the same exercises that the HIFT group did on Friday. In all groups, the main goal of the training was to perform each repetition during the training unit with maximum intensity. The intensity of the exercises was to be greater than 85% HR_max_ and it was monitored using a POLAR m400 sportester *(Kempele, Finland*) and LA concentration 5 min after training.

### Statistical analysis

Statistica version 13.1 (*StatSoft, Cracow, Poland*) was used to perform statistical analysis. All values were presented as a mean ± SD. All analyses of variance for the system with repeated measurements were performed using the ANOVA/MANOVA test and were verified based on normality of the distribution (Shapiro-Wilk) and homogeneity of variance (Levene test). In each case when the Levene test showed non-compliance with the assumption of homogeneity of variance (small groups below 30 observations), the inference was confirmed by nonparametric tests. According to Kruskal-Wallis ANOVA tests, comparisons between 4 groups or Wilcoxon pair order test for tests repeated in each group separately. If the interpretation of differences obtained by both methods was consistent, it was assumed in a given case that the parametric analysis of variance is resistant to failure to meet the assumption of homogeneity of variance. The size of the effects of the observed variables was determined by partial eta square (η^2^). The values of η^2^ between 0.01–0.05 were evaluated as a low effect, 0.06–0.13 as medium effect and above 0.14 as a high effect. The Bonferroni parametric post-hoc test was used to determine the differences between the groups. In all the tests used, a statistically significant level was set at p ≤ 0.05.
